# Meningitis and neurosensory hypoacousia due to *Rickettsia* sp*.* in Peru: case report

**DOI:** 10.17843/rpmesp.2024.413.13393

**Published:** 2024-08-27

**Authors:** Mario J. Agramonte, Stalin Vilcarromero, Zoila Núñez-Melgar

**Affiliations:** 1 Hospital Nacional Edgardo Rebagliati Martins, Lima, Peru Hospital Nacional Edgardo Rebagliati Martins Lima Peru

**Keywords:** Meningitis, Rickettsia Infections, Hearing Loss, Sensorineural

## Abstract

We present the case of a young female health worker, resident in a high Andean region of Peru, with recent exposure to farm animals and arthropods, who developed acute febrile undifferentiated syndrome, severe thrombocytopenia and pulmonary and abdominal extravasation. Subsequently, the patient developed meningitis and early onset bilateral neurosensorial hypoacusis and showed reactive serology to acute infection by Rickettsia sp. Epidemiological and clinical considerations in the differential diagnosis for early management are discussed.

## INTRODUCTION

Rickettsioses are a group of zoonoses caused by obligate intracellular bacteria of the family *Rickettsiae* sp. They are distributed worldwide and are transmitted by arthropods such as ticks, lice, mites and fleas. The classic manifestation is an acute febrile syndrome, predominantly with thrombocytopenia ^1^^,^^2^, with or without necrotic eschar at the inoculum site, with or without rash and exceptionally with severe manifestations of the nervous system.

A seroprevalence study in the Peruvian Amazon found IgG antibodies in 43.6% in its population for spotted fever and 10.3% for typhus ^3^^,^^4^. In high Andean areas, the prevalence of antibodies for rickettsiosis is up to 47.6% ^5^. Since 2018, cases of *Rickettsia asembonensis* infection have been reported in fleas of domestic animals and in people with nonspecific febrile syndrome in tropical and high Andean areas of Peru ^6^^,^^7^.

We present the case of a woman from the high Andean region of Peru with fever, lumbago, thrombocytopenia, intense headache and hypoacusis associated with infection by *Rickettsia* sp.

## CASE REPORT

A 29-year-old female patient, laboratory technician, with no pathologic medical history, but with epidemiologic history of frequent trips to the high Andean region of Huamanga in Ayacucho and exposure to stray dogs. The patient went to a health facility in Huamanga with low back pain and headache, which started the day before. She was prescribed symptomatic treatment and returned the following day with persistent symptoms and a sensation of high temperature. Blood tests at that time (day 3 of illness) are described in table 1. She was instructed to continue with symptomatic treatment, but two days later she returned with more intense symptoms and was hospitalized. The symptoms were holocranial headache of moderate to severe intensity, photophobia and decreased hearing, besides myalgia in the lumbosacral region and lower limbs, vomiting and oral intolerance.

**Table 1:** Laboratory results.

	Day 2 ^a^	Day 3 ^a^	Day 7 ^a^ (hospital)	Day 8 ^a^ (hospital)	Day 9 ^a^ (hospital)	Day 10 (hospital)	Day 1 with doxycycline (hospital)	Day 6 with doxycycline (hospital)	Day 10 with doxycycline (discharge)
Leucocytes	10,790	12,600	17,000	15,400	13,400	10,480	8570	8420	7750
Neutrophiles (total)	8200	-	-	-	-	8370	5990	-	5600
Platelets (cel/uL)	171,000	159,000	92,000	27,000	40,000	165,000	95,000	334,000	443,000
PT/APTT	-	52 / 102	-	9 / 24	14 / 39	11 / 34	13 / 35	13 / 31	-
CPR	-	-	25	76			17	6.8	6.9
TGO / TGP	-	-	-	-	-	13 / 25	13 / 18	21 / 32	-
Creatinine / urea	-	-	1.3 / 51	-	0.92 / 47	0.52 / 41	0.38 / 23	-	0.41 / 35
BT / BI	-	-	-	2.7 / 2	-	-	1.5 / 1,05	1.05 / 0.5	-

a Days since onset of illness.

PT/APTT: prothrombin time/activated partial thromboplastin time, CRP: C-reactive protein, LDH: lactate dehydrogenase, TGO/TGP glutamic oxaloacetic transaminase/glutamic pyruvic transaminase, BT/BI: total bilirubin/indirect bilirubin.

A CT scan of the brain showed diffuse cortical atrophy with absence of hemorrhagic, ischemic or expansive lesions. Chest tomography showed no pathological findings. Blood tests at hospital admission on day 7 of illness showed leukocytosis, increased CRP, and thrombocytopenia (Table 1). Bacterial meningitis, leptospirosis and dengue were considered as differential diagnosis, then treatment with ceftriaxone, amikacin and dexamethasone started. However, on day 8 of illness, due to persistent thermal elevation (>38 °C) and decrease in platelet count (down to 27 000/uL), she was transferred to a referral hospital in Lima for further diagnostic studies.

### Clinical findings

On admission to our hospital, physical examination revealed a patient in fair general condition, with mild generalized pallor, bilateral eyelid edema and a very mild maculo-erythematous rash on the neck and thorax.

**Figure 1:**
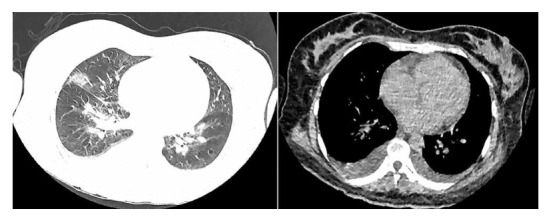
Chest CT scan without contrast with bilateral pleural effusion that resolved in the convalescent phase, no lesions in lung parenchyma.

**Figure 2:**
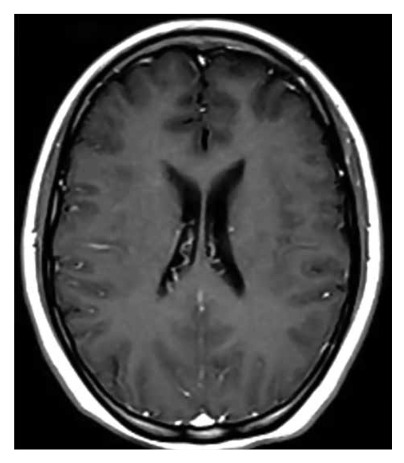
Brain tomography, without acute alterations.

During neurological evaluation the patient was awake, alert, oriented in person, space and time, muscle strength and sensitivity were preserved, Kernig and Brudzinki sign was negative, nuchal rigidity (+), bilateral Lasegue (+) and bilateral hypoacusis. In addition, the patient presented mild abdominal pain on deep palpation, decreased vesicular murmur at the base of the right hemithorax, oxygen saturation of 97% with room oxygen. A new chest CT scan was requested, which showed a predominantly right pleural effusion of small volume, with no alterations in the lung parenchyma or mediastinum (Figure 1). A brain MRI was also performed, which showed no alterations (Figure 2).

### Diagnostic evaluation

A lumbar puncture was performed and cerebrospinal fluid (CSF) was obtained with clear appearance, opening pressure of 22 cm H_2_0, with a cell count of 6 cells/uL, predominance of lymphocytes, hypoglycorrhachia (10 mg/dL for a glycemia by hemoglucotest of 81 mg/dL), hyperproteinorrachia (211 mg/dL), adenosine deaminase at 3UI, with negative culture for common germs and fungi.

GenXpert for tuberculous mycobacteria in CSF was not performed. Serological studies for dengue (Elisa NS1), leptospirosis (Elisa IgM), brucellosis (tube agglutination and 2-mercaptoethanol) were performed with non-reactive results. Indirect immunofluorescence for IgM and IgG antibodies against *Rickettsia* sp. performed at the National Institute of Health was positive (titer of 1:256 for IgM). The medical team decided to continue with empirical antibiotic coverage for bacterial meningitis for five days, with the expectation of empirical initiation of antituberculosis treatment and/or a new CSF study depending on the clinical evolution. The positive serology for rickettsiae, the discrete improvement of the clinical picture after starting doxycycline, and the absence of other risk factors for tuberculous disease allowed us to conservatively evaluate the patient.

### Therapeutic intervention

Treatment with doxycycline 100 mg orally, every 12 hours, continued for nine days and a marked improvement of the headache was evidenced. After 15 days of hospitalization, laboratory parameters normalized (Table 1). The headache and other symptoms subsided, with the exception of bilateral hypoacusis. The patient was discharged from the hospital.

Two weeks after discharge, an otorhinolaryngological evaluation with audiometry was performed and concluded with the diagnosis of right profound sensorineural hearing loss and left moderate-severe sensorineural hearing loss.

## DISCUSSION

Rickettsial diseases are characterized by an acute febrile syndrome and headache, arthralgias, myalgias and other nonspecific symptoms, accompanied or not by a rash usually maculopapular, although sometimes mild papulovesicular or intense petechial rash. There may also be an eschar at the inoculation site ^8^^,^^9^ and involvement of other organs and systems. Our patient presented rash and an acute febrile syndrome; a picture that has also been described in cases with *Rickettsia* sp. in Peru ^8^^,^^9^. The usually maculopapular rash has been described in up to 42% of febrile patients with positive serology for rickettsiae of the Spotted Fever Group (SFG) in Peru, while it has been less frequent in some outbreaks caused by rickettsiae of the Typhus Group (TG) ^10^. Other reported manifestations include thrombocytopenia and pleural effusion ^11^^,^^12^, which were present in our patient and resolved during convalescence.

This initial febrile presentation in our patient was indistinguishable from other vector-borne febrile syndromes, however, her epidemiological history (high Andean region and contact with animals) added to the presence of thrombocytopenia placed rickettsiosis as a differential diagnosis ^1^. Another option was leptospirosis, however, in Peru, it is more frequently reported in regions with tropical climates such as Loreto or Madre de Dios ^13^ and, in addition, serology tests performed after five days ruled out this infection.

Involvement of the central nervous system is rarely caused by rickettsiosis, such as aseptic meningitis or involvement of the auditory nerve ^12^^,^^14^. The clinical picture and CSF study of our patient corresponded to aseptic meningitis in the context of an acute *Rickettsia* sp. infection ^2^. Sensorineural hearing loss has been reported as a complication due to infection by SFG or TG rickettsiae, although in a lower proportion in the latter group ^14^^-^^16^. Rossio *et al*. report a patient with classic Mediterranean spotted fever, produced by *Rickettsia conorii* (SFG), who at the end of the acute phase, similar to what happened to our patient, developed hearing loss, without recovery until the third month of follow-up ^16^. On the other hand, Tsiachris *et al*. describe this complication in an infection by *Rickettsia tiphy* (TG), during convalescence (second or third week of illness, after starting treatment) and with recovery of hearing 3 to 4 weeks after the end of treatment ^14^.

It is possible that this complication is due to a secondary immune reaction ^14^. Although, it has also been described by direct invasion of rickettsiae in the central nervous system, due to vasculitis during the acute phase, affecting structures such as the eighth cranial nerve. The latter would explain the early appearance of this complication in our patient ^15^^,^^16^. It should be mentioned that exposure to aminoglycosides could have contributed to sensorineural deafness in the patient and in this case, the two mechanisms of cochlear lesion would be due to exposure to standard doses of these drugs or genetic predisposition to it ^17^^,^^18^. Another possibility would be secondary to meningitis, however, the early onset of hearing loss and management with corticosteroids would exclude this possibility. The clinical and audiometric follow-up performed one month after discharge would show no improvement, which would indicate irreversible damage.

Regarding etiological diagnosis, in Peru, confirmation of cases of rickettsiosis is performed by the National Institute of Health using the indirect immunofluorescence technique (IIF), which allows detection of IgG, IgM antibodies or both, but with the limitation of not differentiating whether the infections are due to SFG or TG rickettsiae. An increase of the serological titer to four times the value obtained in the first sample or a titer ≥ 1:256 in the acute stage, as happened in our case, is considered positive ^11^^,^^12^. Molecular studies could not be performed to identify the species involved, but due to TG frequency it would be the most probable group in this high Andean area. It should be mentioned that *Rickettsia asembonensis* (SFG) has also been reported in this region, but its clinical spectrum is not completely elucidated ^4^^,^^7^. Palacios-Salvatierra *et al*. identified the presence of *Rickettsia felis* in Tacna and Candidatus Rickettsia asemboensis in Loreto, Madre de Dios, Ayacucho and Cajamarca ^4^^,^^7^. However, not all rickettsial species circulating in Peru have been characterized due to the difficulty of microbiological or molecular diagnosis in the regions ^7^.

The serological laboratory diagnosis is one of the limitations of our report, however, the high titers at the time of diagnosis and the negative control obtained weeks later, added to the resolution of the clinical picture and laboratory parameters, are sufficient evidence of acute infection. In addition, at the time this patient was admitted, our institution did not have the GenXpert MTB Rif platform and although an attempt was made, it was not possible to arrange the test in other entities due to the economic limitations of our patient.

Early initiation of treatment with doxycycline is recommended to reduce the occurrence of complications, especially in severe forms of disease, at doses of 100 mg every 12 h ^19^^,^^20^. However, doses of 200 mg (as a loading dose on the first day) as well as combination therapies have been tested for some forms of rickettsiosis ^19^^,^^21^. Varghese *et al*. in a randomized clinical trial concluded that intravenous administration of doxycycline plus azithromycin was superior to monotherapy, with fewer complications at day 7 and lower mortality at 28 days ^21^.

The availability of the serological results, the positive result in the first reading for rickettsiae and the suggestion to start early doxycycline in case of suspicion of severe disease due to *Rickettsia* sp. ^19^^,^^20^ inclined the medical discussion not to start empirical antituberculosis treatment, unless the patient’s evolution was poor.

In conclusion, rickettsial infections should be considered as differential diagnosis for all patients with acute febrile syndrome and exposure to arthropods or related animals, plus thrombocytopenia, coming from the high Andean or Amazonian regions. In addition, the possibility of meningitis and/or involvement of the VIII cranial nerve should be considered in the presence of alarm signs such as intense headache or hypoacusis, and early management with doxycycline should be initiated.

Author contributions. All authors declare that they meet the authorship criteria recommended by the ICMJE.

## References

[1] Tomassone L, Portillo A, Nováková M, de Sousa R, Oteo JA (2018). Neglected aspects of tick-borne rickettsioses. Parasit Vectors.

[2] Sekeyová Z, Danchenko M, Filipcík P, Fournier PE (2019). Rickettsial infections of the central nervous system. PLOS NTDs.

[3] Forshey BM, Stewart A, Morrison AC, Gálvez H, Rocha C, Astete H (2010). Epidemiology of spotted fever group and typhus group rickettsial infection in the Amazon basin of Peru. Am J Trop Med Hyg.

[4] Palacios-Salvatierra R, Anaya-Ramírez E, Juscamayta-López J, Cáceres-Rey O, Mendoza-Uribe L, Mosquera-Visaloth P (2017). Perfil epidemiológico y molecular de Rickettsiosis en localidades de frontera peruana. Rev Perú Med Exp Salud Publica.

[5] Salmon-Mulanovich G, Simons MP, Flores-Mendoza C, Loyola S, Silva M, Kasper M (2019). Seroprevalence and risk factors for rickettsia and leptospira infection in Four Ecologically Distinct Regions of Peru. Am J Trop Med Hyg.

[6] Kocher C, Morrison AC, Leguia M, Loyola S, Castillo RM, Galvez HA (2016). Rickettsial Disease in the Peruvian Amazon Basin. PLOS NTDs.

[7] Palacios-Salvatierra R, Cáceres-Rey O, Vásquez-Domínguez A, Mosquera-Visaloth P, Anaya-Ramírez E (2018). Especies rickettsiales en casos humanos con síndrome febril agudo inespecífico en Perú. Rev Perú Med Exp Salud Publica.

[8] Abarca K, Oteo JA (2014). Clinical approach and main tick-borne rickettsiosis present in latin america. Rev Chil Infectol.

[9] Faccini-Martínez ÁA, Forero-Becerra EG, Cortés-Vecino JA, Polo-Teran LJ, Jácome JH, Vargas JJ (2013). Caso probable de fiebre manchada (Rickettsia felis) transmitida por pulgas. Biomedica.

[10] Mostorino E Rosa, Anaya R Elizabeth, Mendoza U Leonardo, Rosas A Angel (2003). Identificación de una nueva área de infección por rickettsias del grupo typhi estudio de un brote de tifus en Huánuco. Rev Peru Med Exp Salud Publica.

[11] Oteo JA, Nava S, de Sousa R, Mattar S, Venzal JM, Abarca K (2014). Guías latinoamericanas de la RIICER para el diagnóstico de las rickettsiosis transmitidas por garrapatas. Rev Chil Infectol.

[12] Botelho-Nevers E (2014). Rickettsiosis y ehrlichiosis. EMC Dermatol.

[13] Ministerio de Salud (2020). Leptospirosis-Reporte Semana Epidemiológica Número 18.

[14] Tsiachris D, Deutsch M, Vassilopoulos D, Zafiropoulou R, Archimandritis AJ (2008). Sensorineural hearing loss complicating severe rickettsial diseases Report of two cases. Journal of Infection.

[15] Kang JI, Kim DM, Lee J (2009). Acute sensorineural hearing loss and severe otalgia due to scrub typhus. BMC Infect Dis.

[16] Rossio R, Conalbi V, Castagna V, Recalcati S, Torri A, Coen M (2015). Mediterranean spotted fever and hearing impairment A rare complication. Int J Infecto Dis.

[17] Rodríguez-Álvarez M (2002). Aminoglucósidos. Enferm Infecc Microbiol.

[18] Quintero Noa Julianis, Hernández Cordero María del Carmen, de León Ojeda Norma Elena, Meléndez Quintero Loraine (2018). Ototoxicidad y factores predisponentes. Rev Cubana Pediatr.

[19] Biggs HM (2016). Diagnosis and Management of Tickborne Rickettsial Diseases: Rocky Mountain Spotted Fever and Other Spotted Fever Group Rickettsioses, Ehrlichioses, and Anaplasmosis - United States. MMWR Recomm Rep.

[20] Blanton LS (2019). The Rickettsioses A Practical Update. Infect Dis Clin North Am.

[21] Varghese GM, Dayanand D, Gunasekaran K, Kundu D, Wyawahare M, Sharma N (2023). Intravenous Doxycycline, Azithromycin, or Both for Severe Scrub Typhus. N Engl J Med.

