# Supplementary material for: Meningitis and neurosensory hypoacousia due to Rickettsia sp. in Peru: case report
Source: Rev Peru Med Exp Salud Publica. 2024 Aug 27;41(3):316–20. doi: 10.17843/rpmesp.2024.413.13393 (PMC11495936; doi:10.17843/rpmesp.2024.413.13393)
Supplement: Translation

# Translation: Meningitis e hipoacusia neurosensorial por *Rickettsia* sp. en Perú: reporte de caso

**Authors:** Mario J. Agramonte, Stalin Vilcarromero, Zoila Núñez-Melgar

**Affiliations:** 1 Hospital Nacional Edgardo Rebagliati Martins, Lima, Perú.

**Keywords:** Meningitis, Infecciones por Rickettsia, Pérdida Auditiva Sensorineural

## Abstract

Se presenta el caso de una mujer joven, trabajadora de salud, residente en una región altoandina del Perú, con exposición reciente a animales de granja y artrópodos, que presenta síndrome febril agudo indiferenciado, trombocitopenia severa y extravasación pulmonar y abdominal. Posteriormente desarrolla meningitis e hipoacusia neurosensorial bilateral de instalación temprana y muestra serología reactiva a infección aguda por *Rickettsias* sp. Se discuten las consideraciones epidemiológicas y clínicas en el diagnóstico diferencial para un manejo oportuno.

## INTRODUCCIÓN

Las rickettsiosis son un grupo de zoonosis producidas por bacterias intracelulares obligatorias de la familia *Rickettsiae* sp*.* Tienen distribución mundial y son transmitidas por artrópodos como garrapatas, piojos, ácaros y pulgas. La manifestación clásica es un síndrome febril agudo, predominantemente con trombocitopenia ^1^^,^^2^, con o sin escara necrótica en el sitio de inóculo, con o sin rash y de manera excepcional con manifestaciones graves del sistema nervioso.

Un estudio de seroprevalencia en la Amazonía peruana encontró anticuerpos IgG en 43,6% para fiebres manchadas y 10,3% para tifus ^3^^,^^4^. En zonas altoandinas, la prevalencia de anticuerpos para rickettsiosis es de hasta 47,6% ^5^. Desde el 2018, se han reportado casos de infección por *Rickettsia asembonensis* en pulgas de animales domésticos y en personas con síndrome febril inespecífico en áreas tropicales y altoandinas del Perú ^6^^,^^7^.

Presentamos el caso de mujer procedente de la región altoandina del Perú con fiebre, lumbalgia, trombocitopenia, cefalea intensa e hipoacusia asociadas a la infección por *Rickettsia* sp*.*

## REPORTE DE CASO

Paciente mujer de 29 años, de profesión técnica de laboratorio, sin antecedentes médicos patológicos, con antecedente epidemiológico de viajes frecuentes a la región altoandina de Huamanga en Ayacucho y con exposición a perros callejeros. La paciente acude a un establecimiento de salud de Huamanga con lumbalgia y cefalea de un día de evolución. Le indican terapia sintomática, regresa al día siguiente por persistencia de síntomas y con sensación de alza térmica. La analítica sanguínea en ese momento (día 3 de enfermedad) se describe en la tabla 1. Le indican continuar con tratamiento sintomático, pero dos días después regresa por mayor intensidad de los síntomas y es hospitalizada. Los síntomas reportados fueron cefalea holocraneana de intensidad moderada a severa, fotofobia y disminución de la audición. Además, mialgias en región lumbosacra y miembros inferiores, vómitos e intolerancia oral.

**Tabla 1:** Resultados de laboratorio.

	Día 2 ^a^	Día 3 ^a^	Día 7 ^a^ (hospital)	Día 8 ^a^ (hospital)	Día 9 ^a^ (hospital)	Día 10 (hospital)	Día 1 con doxiciclina (hospital)	Día 6 con doxiciclina (hospital)	Día 10 con doxiciclina (alta)
Leucocitos	10 790	12 600	17 000	15 400	13 400	10 480	8570	8420	7750
Neutrófilos (total)	8200	-	-	-	-	8370	5990	-	5600
Plaquetas (cel/uL)	171 000	159 000	92 000	27 000	40 000	165 000	95 000	334 000	443 000
TP / TTPa	-	52 / 102	-	9 / 24	14 / 39	11 / 34	13 / 35	13 / 31	-
PCR	-	-	25	76			17	6,8	6,9
TGO / TGP	-	-	-	-	-	13 / 25	13 / 18	21 / 32	-
Creatinina / urea	-	-	1,3 / 51	-	0,92 / 47	0,52 / 41	0,38 / 23	-	0,41 / 35
BT / BI	-	-	-	2,7 / 2	-	-	1,5 / 1,05	1,05 / 0,5	-

a Días trascurridos desde inicio de la enfermedad.

TP/TTPa: tiempo de protrombina/tiempo de tromboplastina parcial activada, PCR: proteína C reactiva, LDH: lactato deshidrogenasa, TGO/TGP transaminasa glutámico oxalacética/transaminasa glutámico pirúvica, BT/BI: bilirrubina total/bilirrubina indirecta.

Le solicitan una tomografía cerebral donde se aprecia atrofia cortical difusa con ausencia de lesiones hemorrágicas, isquémicas o expansivas. La tomografía de tórax no muestra hallazgos patológicos. La analítica sanguínea de ingreso hospitalario en el día 7 de enfermedad muestra leucocitosis, PCR incrementada, y trombocitopenia (Tabla 1). Se considera a la meningitis bacteriana, leptospirosis y dengue en el diagnóstico diferencial e inician terapia con ceftriaxona, amikacina y dexametasona. Sin embargo, en el día 8 de enfermedad, debido a la persistencia del alza térmica (>38 °C) y el descenso del recuento de plaquetas (hasta 27 000/uL), es trasladada a un hospital de referencia en Lima, para mayores estudios de diagnóstico.

### Hallazgos clínicos

Al ingreso en nuestro hospital, en el examen físico, se aprecia una paciente en regular estado general, con palidez leve y generalizada, edema palpebral bilateral y un rash maculo eritematoso muy leve en cuello y tórax.

En la evaluación neurológica la paciente se encuentra despierta, alerta, orientada en persona, espacio y tiempo, fuerza muscular y sensibilidad conservadas, signo de Kernig y Brudzinki negativos, rigidez de nuca (+), Lasegue bilateral (+) e hipoacusia bilateral. Además, dolor abdominal leve a palpación profunda, disminución del murmullo vesicular en base de hemitórax derecho, saturación de oxigeno de 97% con oxígeno ambiental. Se solicita una nueva tomografía de tórax donde se evidencia derrame pleural de predominio derecho de escaso volumen, sin alteraciones en el parénquima pulmonar o el mediastino (Figura 1). Se realizó además una resonancia cerebral que no mostró alteraciones (Figura 2).

**Figura 1:** Tomografía de tórax sin contraste con efusión pleural bilateral que se resolvió en la fase convaleciente, no lesiones en parénquima pulmonar.Figura 2. Tomografía de cerebro, sin alteraciones agudas.

**Figura 2:** Tomografía de cerebro, sin alteraciones agudas.

### Evaluación diagnóstica

Se realiza una punción lumbar y se obtiene líquido cefalorraquídeo (LCR) de aspecto claro, presión de apertura de 22 cm H_2_0, con recuento celular de 6 células/uL, predominio de linfocitos, hipoglucorraquia (10 mg/dL para una glicemia por hemoglucotest de 81 mg/dL), hiperproteinorraquia (211 mg/dL), adenosina deaminasa en 3UI, con cultivo negativo para gérmenes comunes y hongos.

No se realizó GenXpert para micobacterias tuberculosas en LCR. Se efectuaron estudios serológicos para dengue (Elisa NS1), leptospirosis (Elisa IgM), brucelosis (aglutinación en tubo y 2-mercaptoetanol) con resultados no reactivos. La inmunofluorescencia indirecta para anticuerpos IgM e IgG contra *Rickettsia* sp*.* realizado en el Instituto Nacional de Salud, resultó positivo (título de 1:256 para IgM). El equipo médico decidió continuar con cobertura antibiótica empírica para meningitis bacteriana por cinco días, con la expectativa de iniciar empíricamente el tratamiento antituberculoso y/o un nuevo estudio de LCR según la evolución clínica. La serología positiva para rickettsias, la mejoría discreta del cuadro clínico tras el inicio de doxiciclina, y la ausencia de otros factores de riesgo para enfermedad tuberculosa nos permitió una valoración conservadora de la paciente.

### Intervención terapéutica

El tratamiento con doxiciclina 100 mg vía oral, cada 12 horas, continuó por nueve días y se evidenció mejoría marcada de la cefalea. Luego de 15 días de hospitalización, los parámetros de laboratorio, se normalizaron (Tabla 1). La cefalea y otros síntomas remitieron, con excepción de la hipoacusia bilateral. Se procedió con el alta médica de la paciente.

Dos semanas después del alta, se lleva a cabo una evaluación otorrinolaringológica con audiometría y se concluye con el diagnostico de hipoacusia neurosensorial profunda derecha e hipoacusia neurosensorial moderada-severa izquierda.

## DISCUSIÓN

Las enfermedades por rickettsias se caracterizan por un síndrome febril agudo y cefalea, artralgias, mialgias y otros síntomas inespecíficos, acompañados o no de un rash usualmente maculopapular, aunque en ocasiones, papulovesicular leve o petequial intenso. También puede presentarse una escara en el lugar de inoculación ^8^^,^^9^ y compromiso de otros órganos y sistemas. Nuestra paciente presentó rash y un síndrome febril agudo; cuadro que también se ha descrito en los casos con *Rickettsia* sp*.* en Perú ^8^^,^^9^. El rash usualmente maculopapular ha sido descrito hasta en un 42% en el caso de pacientes febriles con serología positiva para las rickettsias del Grupo de las Fiebre Manchada (Spotted Fever Group o SFG) en el Perú, mientras que ha sido menos frecuente en algunos brotes causados por rickettsias del Grupo Tiphy (Tifus Group o TG) ^10^. Otras manifestaciones reportadas incluyen trombocitopenia y efusión pleural ^11^^,^^12^, que estuvieron presentes en nuestra paciente y que se resolvieron en el periodo de convalecencia.

Esta presentación febril inicial en nuestro paciente es indistinguible de otros síndromes febriles transmitidos por vectores, sin embargo, el antecedente epidemiológico mencionado (región altoandina y contacto con animales) sumado a la presencia de trombocitopenia colocó a la rickettsiosis en el diagnóstico diferencial ^1^. Otra opción era leptospirosis, sin embargo, en el Perú, es más frecuentemente reportado en regiones con climas tropicales como Loreto o Madre de Dios ^13^ y, además la serología de la muestra obtenida después de los cinco días descartó esta infección.

Rara vez se describe compromiso de sistema nervioso central por rickettsiosis, como meningitis aséptica o compromiso del nervio auditivo ^12^^,^^14^. El cuadro clínico y estudio de LCR de nuestra paciente correspondió con meningitis aséptica en el contexto de una infección aguda por *Rickettsia* sp. ^2^. Con respecto a la hipoacusia neurosensorial, se ha reportado como complicación debido a la infección por las rickettsias del SFG o del TG, aunque en menor proporción en este último grupo ^14^^-^^16^. Rossio *et al.* reportan un paciente con fiebre manchada mediterránea clásica, producida por *Rickettsia conorii* (SFG), que al final de la fase aguda, similar a lo ocurrido a nuestro paciente, desarrolló pérdida auditiva, sin recuperación hasta el tercer mes de seguimiento ^16^. Por otro lado, Tsiachris *et al.* describen esta complicación en una infección por *Rickettsia tiphy* (TG), durante la convalecencia (segunda o tercera semana de enfermedad, después de iniciado el tratamiento) y con recuperación de la audición 3 a 4 semanas después de terminado el tratamiento ^14^.

Es posible que esta complicación sea debido a una reacción inmune secundaria ^14^. Aunque, también se ha descrito por invasión directa de las rickettsias en el sistema nervioso central, debido a la vasculitis durante la fase aguda, que afecta estructuras como el octavo par craneal. Esto último explicaría la aparición temprana de ésta complicación en nuestra paciente ^15^^,^^16^. Debe mencionarse que la exposición a aminoglucósidos pudo haber contribuido con la sordera neurosensorial en la paciente y en ese caso, los dos mecanismos de lesión coclear serian por exposición a dosis estándar de estos fármacos o, por predisposición genética a la misma ^17^^,^^18^. Otra posibilidad sería secundaria a la meningitis, sin embargo, el inicio precoz de la hipoacusia y del manejo con corticoides alejarían esta posibilidad. El seguimiento clínico y de audiometría realizado al mes del alta no mostraría mejoría, lo que indicaría el daño irreversible.

Con respecto al diagnóstico etiológico, en el Perú, la confirmación de los casos de rickettsiosis lo realiza el Instituto Nacional de Salud usando la técnica de inmunofluorescencia indirecta (IFI) que permite detectar anticuerpos IgG, IgM o ambas, pero con la limitación de no diferenciar si las infecciones son por rickettsias del SFG o TG. Se considera positivo un aumento del título serológico al cuádruple del valor obtenido en la primera muestra o un título ≥ 1:256 en la etapa aguda como sucedió en nuestro caso ^11^^,^^12^. No se pudieron realizar estudios moleculares, para identificar la especie involucrada, pero por frecuencia TG sería el grupo más probable en esta zona altoandina. Debe mencionarse que *Rickettsia asembonensis* (SFG) ha sido reportado también en esta región, pero su espectro clínico no está completamente dilucidado ^4^^,^^7^. Palacios-Salvatierra *et al.* identificaron la presencia de *Rickettsia felis* en Tacna y *Candidatus Rickettsia asemboensis* en Loreto, Madre de Dios, Ayacucho y Cajamarca ^4^^,^^7^. Sin embargo, no se han caracterizado todas las especies de rickettsias que circulan en Perú debido a la dificultad para el diagnóstico microbiológico o molecular en las regiones ^7^.

Dentro de las limitaciones del presente reporte se debe mencionar que el diagnóstico de laboratorio es serológico, sin embargo, los títulos altos al momento de diagnóstico y el control negativo obtenido semanas después, sumado a la resolución del cuadro clínico y parámetros de laboratorio, son suficiente evidencia de la infección aguda. Además, al momento en que se atendió a esta paciente nuestra institución no contaba con la plataforma GenXpert MTB Rif y aunque se intentó, no se logró gestionar la prueba en otras entidades debido a las limitaciones económicas de nuestra paciente.

Se recomienda el inicio temprano del tratamiento con doxiciclina, para reducir la ocurrencia de complicaciones, sobre todo en formas severas de enfermedad, a dosis de 100 mg cada 12 h ^19^^,^^20^. Sin embargo, se han ensayado dosis de 200 mg (como dosis de carga el primer día) así como terapias combinadas para algunas formas de rickettsiosis ^19^^,^^21^. Varghese *et al.* en un ensayo clínico aleatorizado, concluyeron que la administración endovenosa de doxiciclina más azitromicina fue superior a la monoterapia, con menores complicaciones al día 7 y menor mortalidad a los 28 días ^21^.

La disponibilidad de los resultados serológicos, el resultado positivo en primera lectura para rickettsias y la sugerencia de iniciar tempranamente doxiciclina ante la sospecha de enfermedad severa por *Rickettsia* sp.^19^^,^^20^ inclinó la discusión médica para no iniciar tratamiento antituberculoso empírico, salvo mala evolución de la paciente.

En conclusión, en todo paciente con síndrome febril agudo con exposición a artrópodos o animales relacionados, más trombocitopenia, procedentes de las regiones altoandinas o amazónicas, debe incluirse a las rickettsiosis en el diagnóstico diferencial. Además, ante la presencia de signos de alarma como cefalea intensa o hipoacusia, debe considerarse la posibilidad de meningitis y/o compromiso del VIII par craneal, e iniciar manejo temprano con doxiciclina.
